# Persistent response of furmonertinib plus anlotinib in a lung adenocarcinoma patient with an EGFR exon 20 insertion mutation: A case report

**DOI:** 10.3389/fphar.2023.1053805

**Published:** 2023-02-03

**Authors:** Xuesong Chen, Wangjian Zha, Mei Su, Nan Meng, Shuliang Cao, Beifang Niu, Xu Qi

**Affiliations:** ^1^ Department of Pulmonary and Critical Care Medicine, The First Affiliated Hospital with Nanjing Medical University, Nanjing, China; ^2^ ChosenMed Technology (Beijing) Co, Ltd, Beijing, China; ^3^ Computer Network Information Center, Chinese Academy of Sciences, Beijing, China; ^4^ University of the Chinese Academy of Sciences, Beijing, China; ^5^ Jiangsu Shengze Hospital, Suzhou, China

**Keywords:** lung adenocarcinoma, EGFR exon 20 insertions, furmonertinib, anlotinib, case report

## Abstract

Insertions in exon 20 represent the third most common type of *EGFR* mutation following in-frame deletions in exon 19 and the point mutation L858R in exon 21. They are generally associated with primary resistance to EGFR-TKIs. Although mobocertinib and amivantamab were approved for adult patients with non-small cell lung cancer (NSCLC) harboring *EGFR* exon 20 insertion mutations, the efficacy of these two agents was rather moderate. Therefore, other more potent targeted agents are urgently needed. Here, we report a patient with advanced lung adenocarcinoma harboring an *EGFR* exon 20 insertion mutation (NM_005228: exon 20: c.2316_2321dup: p.773_774dup). After experiencing platinum-based chemotherapy, this patient received a combination of furmonertinib and anlotinib and achieved lasting stable disease (SD). The treatment was well tolerated, and only mild hand-foot syndrome was reported from the patient. To the best of our knowledge, this case firstly reported the encouraging efficacy of combined furmonertinib and anlotinib in an advanced lung adenocarcinoma patient with an *EGFR* exon 20 insertion mutation who was previously treated with platinum-based chemotherapy. In addition, we summarize the recent literature on therapies against NSCLC with *EGFR* exon 20 insertion mutations. This case might provide an alternative approach for clinical oncologists.

## Background

Over the past decade, the development of *EGFR* tyrosine kinase inhibitors (TKIs) has revolutionized the therapy of non-small cell lung cancer (NSCLC) with mutated epidermal growth factor receptor (*EGFR*) gene (Zhou et al., 2015; Soria et al., 2018). However, patients with NSCLC harboring *EGFR* exon 20 insertions (*EGFR* ex20ins) mutations, accounting for approximately 10% of all *EGFR*-positive NSCLC cases, usually show *de novo* resistance to the approved *EGFR*-TKIs (Yasuda et al., 2012).

The incidence of *EGFR* p.773_774dup is very low, accounting for approximately 2.22% of *EGFR* ex20ins in China (Shun Xu, 2022). *In vitro* studies showed that compared with other *EGFR* ex20ins, *EGFR* p.773_774dup was the least sensitive to the first-generation or second-generation EGFR-TKIs. Furthermore, a clinical study demonstrated that the overall response rate (ORR) was 0% for the patients with EGFR p.773_774dup mutation, while 17% for the patients with EGFR ex20ins variations when receiving the first-generation EGFR-TKIs (Kobayashi and Mitsudomi, 2016).

Recently, much progress has been made against NSCLCs with *EGFR* ex20ins mutations (Meador et al., 2021). For example, the Food and Drug Administration (FDA) of U.S. approved two novel agents (mobocertinib and amivantamab) for adult patients with NSCLC who harbor *EGFR* ex20ins mutations in 2021. However, the efficacy of these two agents was rather moderate compared with TKIs targeting canonical *EGFR* mutations (Zhou C. et al., 2021; Park et al., 2021; Minchom et al., 2022). Consequently, other more potent therapeutic strategies are urgently needed.

Here, we report a case of pretreated advanced lung adenocarcinoma with *EGFR* ex20ins (NM_005228: exon 20:c.2316_2321dup: p.773_774dup) benefiting from combined furmonertinib, a novel third-generation *EGFR*-TKI, with anlotinib, a novel multi-targeting tyrosine kinase inhibitor, for a long time.

## Case presentation

**FIGURE 1:**
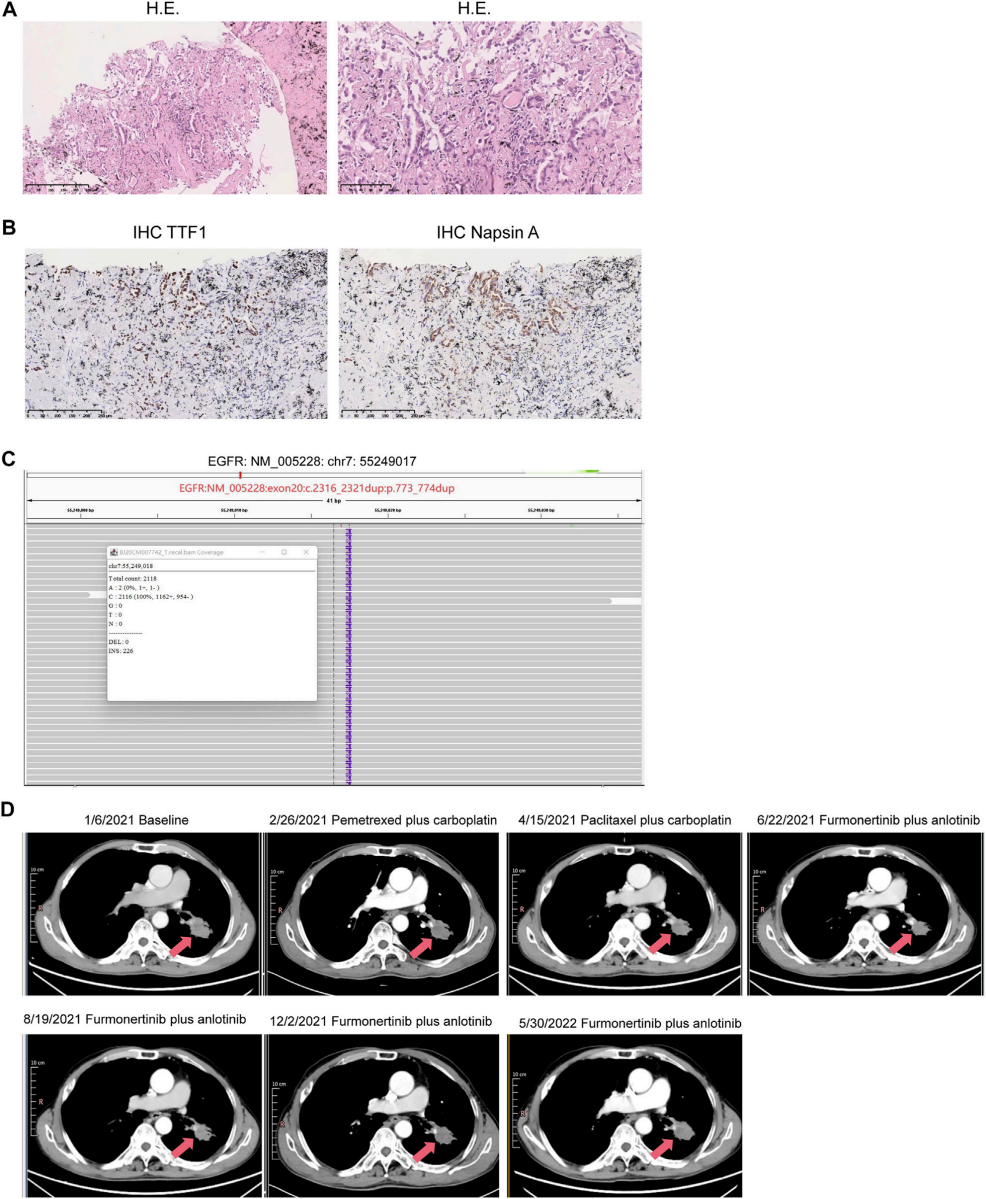
Pathology, the sequencing reads of EGFR p.773_774dup, dynamic imaging of the space-occupying lesion on the left lung during the treatment. **(A)** Pathological findings revealed the lesion on the left lung was moderate-differentiated adenocarcinoma. H.E.: hematoxylin-eosin staining. Scale bars represent 250 μm (left) and 100 μm (right), respectively. **(B)** Immunohistochemistry (IHC) showed positive expression of thyroid transcription factor 1 (TTF1) and Napsin A. Scale bars represent 250 μm (left and right). **(C)** Visualization of EGFR p.773_774dup mutation using the Integrated Genomics Viewer (IGV) browser. **(D)** CT images showed that the patient’s condition remained stable after receiving furmonertinib plus anlotinib. CT: computed tomography.

On 01 January 2021, a 68-year-old male was admitted to a local hospital due to chest and back pain on the left side. The patient had a 30-year smoking history (20 cigarettes per day) and quit it 10 years ago. He had no history of drinking. Thoracic computed tomography (CT) demonstrated a lumpy and hyper-density shadow on the left upper lung, considered a space-occupying lesion. The patient was referred to our department on 5 January 2021 for enhanced multi-slice CT scan, which showed multiple bilateral pulmonary nodules. The largest one was approximately 7 mm in diameter. Furthermore, an irregular soft tissue mass, measuring approximately 33 × 26 mm in size, could be seen in the dorsal segment of the left lower lobe. After contraindications were excluded, CT-guided lung biopsy was performed on 7 January 2021. The pathology confirmed that the lesion on the left lung was moderate-differentiated adenocarcinoma (Figure 1A). Immunohistochemistry (IHC) showed positive expression of thyroid transcription factor 1 (TTF1) and Napsin A (Figure 1B). The final diagnosis was stage IV lung adenocarcinoma (T2aN2M1c). On 12 January 2021, a radionuclide bone scan showed multiple abnormal bone metabolism (data not shown), which might be caused by metastasis. After fully communicating with the patient and his family members, the infusion port implantation was performed on 12 January 2021. Meanwhile, paraffin-embedded sections of tumor tissues from the patient were subjected to next-generation sequencing (NGS) through a 599-gene panel (ChosenMed Technology [Beijing] Co. Ltd., Beijing, China). The results showed that the patient harbored a non-frameshift insertion mutation in *EGFR* (NM_005228: exon 20:c.2316_2321dup: p.773_774dup), with variant allele fraction (VAF) 13.70%. The sequencing reads of *EGFR* are demonstrated in Figure 1C. The somatic alterations in the patient are shown in Table 1.

**TABLE 1:** Somatic variations in the patient.

Gene	Transcript	Exon	Nucleotide change	Alteration	Mutant allele frequency (%)
EGFR	NM_005228	Exon 20	c.2316_2321dup	p.773_774dup	13.70
ARID1A	NM_006015	Exon 19	c.4994–2A>G	-	16.80
BLM	NM_000057	Exon 7	c.1544dup	p.N515fs	3.16
KMT2D	NM_003482	Exon 39	c.11714_11716del	p.Q3905del	2.28
PIK3R2	NM_005027	Exon 13	c.1723G>A	p.D575N	14.60
TMB	2.53 Muts/Mb, ranking among patients with this cancer: 88.29%				
MSI status	MSS				

TMB: tumor mutational burden; MSI: microsatellite instability; MSS: microsatellite stability.

**FIGURE 2:**
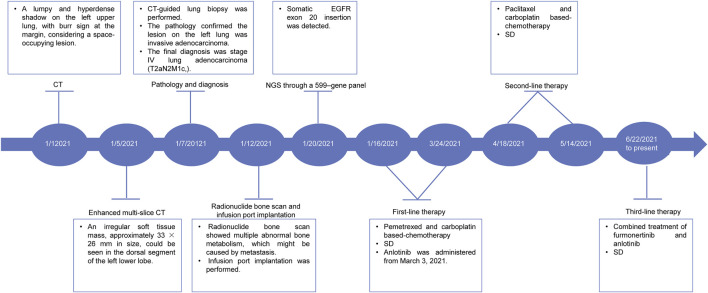
Case timeline.

Subsequently, two cycles of pemetrexed (0.8 g on day 1, repeated every 3 weeks) and carboplatin (0.5 g on day 1, repeated every 3 weeks) based-chemotherapy was administered on January 16 and 6 February 2021. During the third-cycle treatment, the lesion was similar to the previous one, and the patient’s condition was assessed as a stable disease (SD). Therefore, the patient continued to receive another two cycles of the abovementioned chemotherapy on March 2 and 24 March 2021. Moreover, anlotinib (12 mg, once a day) was supplemented from 3 March 2021. After four cycles of treatment, the patient’s condition remained stable. Considering that the lesion did not shrink remarkably, the treatment strategy was switched to two cycles of paclitaxel (0.24 g on day 1, repeated every 3 weeks) and carboplatin (0.5 g on day 1, repeated every 3 weeks) based-chemotherapy on April 18 and 14 May 2021. The patient’s condition was accessed as SD on 22 June 2021. However, the patient refused to continue the chemotherapy because of chest pain. Therefore, targeted therapy with the combination of furmonertinib (160 mg, once a day) and anlotinib (12 mg, once a day) was administered from 22 June 2021. The treatment was well tolerated, and only mild hand-foot syndrome was observed (data not shown). According to the latest follow-up on 30 May 2022, the patient’s condition was evaluated as a SD based on thoracic CT (Figure 1D). The patient achieved a long-term SD after receiving furmonertinib and anlotinib. The case timeline is shown as Figure 2.

## Discussion

To the best of our knowledge, we are the first to describe a case of pretreated advanced lung adenocarcinoma with *EGFR* ex20ins benefiting from the combined furmonertinib and anlotinib for a long time.

Unlike canonical *EGFR* mutations, i.e., *EGFR* 19-del and 21-L858R mutations, patients with *EGFR* ex20ins are not sensitive to most types of EGFR-TKIs and have shorter overall survival (OS) (Oxnard et al., 2013). The underlying mechanism is that *EGFR* ex20ins mutation alters the conformation at the kinase active site, limiting the binding of early-generation EGFR-TKIs (Robichaux et al., 2018; Vyse and Huang, 2019). Over the past few years, the standard therapy of *EGFR* ex20ins has been platinum-based chemotherapy. Recently, two novel agents have been approved for the advanced NSCLC with *EGFR* ex20ins. One is mobocertinib (TAK-788), which is a targeted inhibitor of *EGFR* ex20ins (Gonzalvez et al., 2021). The other is amivantamab (JNJ-61186372), which is a bispecific antibody targeting EGFR and MET (Yun et al., 2020). According to previous studies, the median progression-free survival (PFS) with mobocertinib and amivantamab was 7.3 months, 8.3 months, respectively, and the median OS was 24.0, 22.8 months, respectively (Zhou C. et al., 2021; Park et al., 2021; Minchom et al., 2022) (Table 2). Obviously, the efficacy of these two agents is unsatisfactory. Therefore, other treatment strategies are being explored. For example, a study by Veggel et al. demonstrated the combination of afatinib with cetuximab showed moderate efficacy in patients with *EGFR* ex20ins-positive NSCLC, with a median PFS of 5.3 months (Bianca van Veggel, 2021) (Table 2). In addition, poziotinib is a potent TKI of *EGFR* and *HER2* exon 20 insertion mutants. The investigation (ZENITH20-1) of Le et al. evaluated the efficacy of poziotinib in pretreated patients with NSCLC harboring *EGFR* ex20ins. The results demonstrated that the ORR was 14.8%, and the disease control rate (DCR) was 68.7%, with a median PFS of 4.2 months (Xiuning Le, 2020) (Table 2).

**TABLE 2:** EGFR ex20ins in NSCLC and response to therapy.

Study	Year	Patients	Therapy used	Outcome
Zhou, C. et al. (Zhou et al., 2021a)	2021	114 platinum-pretreated patients with EGFR ex20ins-positive metastatic NSCLC	Mobocertinib	ORR was 28%, median PFS was 7.3 months, and median OS was 24.0 months
Park, K. et al. (Park et al., 2021)	2021	81 patients with EGFR ex20ins NSCLC progressing on platinum chemotherapy	Amivantamab	ORR was 40% including 3 CR and median PFS was 8.3 months
Minchom, A. et al. (Minchom et al., 2022)	2022	81 patients with advanced NSCLC harboring EGFR ex20ins mutations who progressed after platinum-based chemotherapy	Amivantamab	ORR was 40%, median PFS was 8.3 months, and median OS was 22.8 months
Veggel et al. (Bianca van Veggel, 2021)	2021	17 patients with advanced NSCLC harboring an EGFR ex20ins mutation	Dual EGFR blockade with afatinib and cetuximab	Median PFS was 5.5 months. Best responses were PR (n = 8), SD (n = 7) or PD (n = 2)
Fang et al. (Fang et al., 2019)	2019	An adenocarcinoma patient with a EGFR ex20ins mutation (M766delinsMATL)	Low-dose afatinib plus cetuximab	PFS was 11.6 months
Le et al. (Xiuning Le, 2020)	2020	115 pretreated patients with NSCLC positive for EGFR ex20ins	Poziotinib	ORR was 14.8%, and DCR was 68.7%. 65% patients had tumor size reductions with a median PFS of 4.2 months
Yasuda, H. et al. (Yasuda et al., 2021)	2021	12 patients with EGFR ex20ins mutation-positive NSCLC	Regular dose (80 mg/day) of osimertinib	None experienced objective response, 7 experienced SD (58.3%), and 5 experienced PD (41.7%). Median PFS was 3.8 months, and the median OS was 15.8 months
Piotrowska et al. (Zofia Piotrowska, 2020)	2020	20 patients with NSCLC harboring EGFR exon 20 insertions	High dose (160 mg/day) of osimertinib	Median PFS was 9.7 months. The best response was PR (n = 4) and CR (n = 1), ORR was 25%; 12 (60%) patients had SD.
Jia et al. (Jia et al., 2022)	2022	An advanced NSCLC patient with EGFR ex20ins	High-dose furmonertinib (160 mg/day)	PFS was 10 months
Jiang et al. (Jiang et al., 2022)	2022	A patient with lung adenocarcinoma harboring EGFR ex20ins (p.P772_H773insV)	high-dose furmonertinib (160–240 mg daily)	The patient achieved PR.

EGFR, ex20ins; EGFR, exon 20 insertion; NSCLC: non-small-cell lung cancer; ORR: overall response rate; PFS: progression-free survival; OS: overall survival; CR: complete remission; PR: partial response; SD: stable disease; PD: progressive disease; DCR: disease control rate.

Meanwhile, scholars also studied the efficacy of third-generation EGFR-TKIs in the patients with NSCLC harboring *EGFR* ex20ins. They found that a regular dose (80 mg/day) of osimertinib has limited efficacy in these patients, with a median PFS of 3.8 months, and a median OS of 15.8 months (Yasuda et al., 2021). However, high dose (160 mg/day) of osimertinib showed promising clinical activity, with a median PFS of 9.7 months (Zofia Piotrowska, 2020) (Table 2). As a novel third-generation EGFR-TKI, furmonertinib (alflutinib/AST2818) has been approved by the National Medical Products Administration (NMPA) of China for pretreated patients with NSCLC harboring an *EGFR* T790M mutation. Moreover, preclinical data demonstrated that furmonertinib had an antitumor effect in Ba/F3 cell line expressing *EGFR* ex20ins, patient-derived xenograft models harboring *EGFR* ex20ins mutation and treatment-naïve NSCLC patients with *EGFR* ex20ins (Baohui Han, 2021; Das et al., 2021). In 2022, Jia et al. reported an advanced NSCLC patient with *EGFR* ex20ins benefited from high-dose furmonertinib (160 mg/day) after progression from mobocertinib, with a PFS of 10 months (Jia et al., 2022) (Table 2). Meanwhile, Jiang et al. also report a patient with lung adenocarcinoma harboring *EGFR* ex20ins who achieved disease control after receiving high-dose furmonertinib (160–240 mg daily, Table 2) (Jiang et al., 2022). Besides, clinical trials like DZD9008 and CLN-081 against *EGFR* exon 20 insertion–positive NSCLC are ongoing (James Chih-Hsin Yang, 2021; Zofia Piotrowska, 2021).

The antitumor activity of furmonertinib is similar to osimertinib in patients with advanced NSCLC positive for *EGFR* T790M mutation. However, the incidence of skin and gastrointestinal disorders with furmonertinib is lower.

Anlotinib is a novel multi-targeting receptor tyrosine kinase (RTK) inhibitor against vascular endothelial growth factor receptor, fibroblast growth factor receptor, platelet-derived growth factor receptors, and c-kit. Previous studies showed anlotinib could inhibit not only tumor angiogenesis, but also tumor cell proliferation (Sebastien Taurin, 2017; Xie et al., 2018). On 8 May 2018, anlotinib was approved as a third-line treatment for patients with advanced NSCLC in China. In addition, anlotinib can remarkably prolong the median PFS in patients with advanced soft tissue sarcoma, medullary thyroid carcinoma and metastatic renal cell carcinoma. Clinical trials have confirmed that anti-angiogenesis agents like bevacizumab and ramucirumab combined with EGFR-TKI could significantly prolong the survival of patients with *EGFR*-mutant NSCLC (Herbst et al., 2011; Nakagawa et al., 2019; Ninomiya et al., 2019; Zhou Q. et al., 2021). The mechanism might be that anti-angiogenic therapy can transiently restore the effective antitumor immunity, normalize the tumor vessels network, and improve drug delivery and efficacy (Jain et al., 2007; Feng et al., 2018). Compared to bevacizumab and ramucirumab, anlotinib is more convenient, as it is orally administered and it can inhibit more targets. Furthermore, several clinical trials have demonstrated the encouraging efficacy and safety of combined anlotinib with EGFR-TKI for previously untreated, *EGFR*-mutated advanced NSCLC patients (Dingzhi Huang, 2020; D. Zhong, 2020; Chu et al., 2022). In addition, a clinical trial of anlotinib combined with furmonertinib is ongoing as the first-line treatment in patients with *EGFR* mutation-positive locally advanced or metastatic NSCLC (NCT04895930). Based on the above, we hypothesized that a combination of anlotinib and furmonertinib could potentially improve the efficacy for *EGFR* ex20ins-positive advanced NSCLC patients. In addition, the anti-angiogenic activity of anlotinib is stronger than that of three other anti-angiogenesis drugs, including sunitinib, sorafenib, and nintedanib, while it has fewer or milder grade 3 or higher adverse effects (Shen et al., 2018).

Of note, although the patient harbored an *ARID1A* mutation (c.4994–2A >G) which might be a positive predictor of immune checkpoint Inhibitor (ICI) therapy (Zhu et al., 2021). However, the mutation in the gene *EGFR* (c.2316_2321dup: p.773_774dup) was supposed to be associated with drug resistance or hyperprogression when receiving ICI therapy (Borghaei et al., 2015; Herbst et al., 2016; Lee et al., 2017; Rittmeyer et al., 2017; Girard et al., 2022). Furthermore, other ICI biomarkers including the low-TMB (2.53 Muts/Mb), MSS status and low PD-L1 expression (22C3: TPS< 1%, CPS< 1) through immunohistochemistry also predict poor clinical outcome of ICI therapy. Therefore, we chose to combine two TKI inhibitors over combination of a TKI with ICI.

To date, this case firstly demonstrates improved efficacy and tolerability of NSCLC patients with *EGFR* ex20ins when receiving furmonertinib combined with anlotinib. However, the underlying mechanism needs to be clarified in the near future.

## Conclusion

To the best of our knowledge, we are the first to report that the combination of furmonertinib and anlotinib is an effective treatment strategy in a patient with *EGFR* ex20ins-positive advanced lung adenocarcinoma who was previously treated with platinum-based chemotherapy. This case might serve as an alternative approach for clinical oncologists.

## Data Availability

The original contributions presented in the study are included in the article/supplementary materials, further inquiries can be directed to the corresponding author.
